# ‘Everywhere I turn, I’m blocked’: a qualitative exploration of experiences of Charles Bonnet syndrome and its impact on physical activity and falls

**DOI:** 10.1093/ageing/afag125

**Published:** 2026-05-17

**Authors:** Katharine Fisher, Caroline Sanders, Jasleen K Jolly, Penelope Stanford, Emma Stanmore

**Affiliations:** Division of Nursing, Midwifery and Social Work/School of Health Sciences, University of Manchester, Manchester, UK; Division of Population Health, Health Services Research and Primary Care/School of Health Sciences, University of Manchester, Manchester, UK; Department of Optometry and Vision Science, University of Melbourne, Melbourne, Victoria, Australia; Jolly Vision Science, Bolton, UK; Division of Nursing, Midwifery and Social Work/School of Health Sciences, University of Manchester, Manchester, UK; Division of Nursing, Midwifery and Social Work/School of Health Sciences, University of Manchester, Manchester, UK

**Keywords:** Charles Bonnet syndrome, falls, physical activity, visual impairment, qualitative research, older adults

## Abstract

**Background:**

Charles Bonnet syndrome (CBS) refers to visual hallucinations that can occur following a decline in vision. Although vision loss increases fall risk and susceptibility to falls-related variables such as activity limitation, the potential contribution of CBS is unclear. The present study adopted a qualitative approach to explore experiences of CBS from different perspectives, including its impact on physical activity and falls.

**Methods:**

Semistructured interviews were conducted with 17 older adults living with CBS (mean age = 76 years) and 12 community falls and sight loss professionals. Reflexive thematic analysis guided data analysis.

**Results:**

Themes represent the ‘CBS journey’ moving from symptom onset to living with CBS. Low CBS awareness, anticipated stigma and professionals’ failure to raise CBS had negative psychological impacts. While walking, recognisable images could evoke a behavioural response and delay insight. Falls occurred when CBS distracted attention from actual surroundings, automatic reactions led to a loss of balance, and panoramic hallucinations caused disorientation and obscured real hazards. CBS also contributed to concern about falling. While moving about, precipitating factors for CBS included fluctuations in light level and situational stress. Common relief techniques were least effective for people with intrusive symptoms. Instead, engaging in enjoyable multisensory activities or low to moderate physical activity for health provided relief.

**Conclusion:**

CBS adds an additional layer of complexity to the aetiology of sight-related falls. To reduce the risk of falls caused by CBS, a multifaceted strategy is indicated that addresses factors at the individual, organisational, educational and health care policy levels.

Key points

- This study provides a unique contribution to the sight loss and falls literature.
- CBS can increase susceptibility to falls and concern about falling.
- When developing therapeutic strategies for CBS, wider impacts such as falls should be considered.
- Clinicians need to be aware of the functional impacts of CBS and sources of support, including Esme’s Umbrella.

## Introduction

As we age, the risk of eye disease increases [1]. Globally, at least 1.2 billion people have irreversible vision loss, with age-related macular degeneration being a leading cause [2]. Following any degree of vision loss, visual hallucinations, known as Charles Bonnet syndrome (CBS), can occur [3]. One in five people with low vision are estimated to be affected by CBS [4], but this is probably conservative, as lack of screening and stigma can preclude disclosure [5]. Predominant theories to explain CBS have some evidential support. Vision loss can cause changes in the excitation–inhibition balance within visual areas of the brain [6, 7] resulting in bursts of hyperexcitability that generate an internal percept (hallucination) [8–10].

Based on patients’ experiences, the nature of CBS appears highly heterogeneous. Content can be simple (patterns, shapes, blobs of colours) or complex (recognisable images, whole scene change) [11] while episode frequency and duration vary [12]. A range of precipitating factors has been reported such as stress [13], inactivity [14] and dim light [15, 16]. Current self-help techniques are mainly reactive (e.g. eye movements), and patients do not always find them helpful [17].

The literature shows that low CBS awareness causes psychological distress as it perpetuates fears about a sinister aetiology [18]. CBS can have physical impacts too. Cox and Ffytche [19] found that visions hindering the ability to carry out daily activities were associated with clinically important ‘negative outcome’ CBS. Existing studies also highlight that CBS can interfere with mobility and cause a fall but do not provide an in-depth understanding of the mechanism of CBS-related falls [20]. Sight loss alone is an established falls risk factor. In addition, the prevalence of falls-related variables such as activity limitation [21] and concern about falling [22] is higher in older adults with vision loss than sighted peers. Interestingly, those factors potentially overlap with exacerbators of CBS including being sedentary [14] and feeling socially disconnected [17]. The contribution of CBS to falls clearly warrants further investigation.

Current knowledge about how CBS impacts physical activity and falls is limited, and CBS awareness amongst professionals supporting older adults with sight loss in the community is not known. To develop meaningful falls prevention recommendations, a holistic, contextualised understanding of CBS from different perspectives is needed. The aim of this study was to explore (i) older adults’ experiences associated with CBS and its impact on physical activity and (ii) the views of community sight loss and falls prevention specialists about CBS, physical activity, and falls.

## Methods

A qualitative design was chosen to enable in-depth exploration of the study topic. Ethical approval was obtained from the University of Manchester research ethics committee (References: 2022-14926-26156/2024-18934-32859). Informed consent was obtained from all participants prior to data collection.

### Eligibility and recruitment

To capture different perceptions, a maximum variation sampling strategy was used (rural or urban location, living status, age, profession). The concept of ‘information power’ [23] informed sample size, guiding reflection about whether the data were sufficient to answer the research question.

#### Participants living with CBS

Community-dwelling adults aged ≥50 years with self-reported eye disease and CBS, who had experienced CBS within the past 12 months and were able to provide informed consent, were eligible to take part. During recruitment, support group leaders at the UK-based charity Esme’s Umbrella acted as gatekeepers, and the study was advertised on their social media. Contact details of interested parties were emailed to K.F., and people responding to the advert made direct contact. Study information was provided in the potential participant’s preferred format (digital, large print). Recontact was made after 48 hours (7 days if sent by post). Any queries about the research were answered, and a date/time for the interview was agreed with people who wished to take part. Immediately before the interview, participants provided verbal consent, which was audio-recorded. Demographic and clinical data were also obtained.

#### Community professionals

Falls and sight loss professionals supporting adults with sight loss aged ≥50 years living in the community were approached to take part. Awareness of CBS was not a prerequisite for participation. An email was sent directly to falls prevention and sensory impairment teams in the North of England, and the study was advertised at a national CBS event to increase geographical reach. Professionals contacted K.F. by email and were then forwarded study information. Those wishing to take part signed a digital consent form, and a date/time for the interview was scheduled.

### Interviews

All participants took part in a semistructured interview with K.F. A distress protocol was developed to support people with CBS if required. The interview schedule for participants with CBS was informed by the literature and K.F.’s professional experience working with older adults with sight loss. Initial open questions were used to encourage participants to share their priorities, followed by focused questions about physical activity and falls. The guide for professionals was based on the literature and findings from people living with CBS. Topics included were screening for CBS, CBS and mobility/falls, precipitating factors and self-management. See Appendix S1 in the supplementary data.

### Analysis

Interviews were transcribed verbatim by K.F., and NVivo version 12 (Lumivero) was used for data management. Each data set (people living with CBS, professionals) was analysed separately. Braun and Clarke’s [24] reflexive thematic analysis (RTA) informed the generation of the themes. RTA was chosen as it is theoretically flexible, suited to experiential research, prioritises inductive analysis and embraces the researcher’s contribution to knowledge. The six phases are (i) familiarisation; (ii) coding; (iii) generate candidate themes; (iv) develop and review themes; (v) refine, define and name themes; and (vi) writing up. K.F. led the analysis but discussed codes, theme development and revisions with E.S. and C.S. [25]. See Appendix S2 in the supplementary data. Strategies to enhance the quality of findings are outlined in Table 1.

**Table 1:** Strategies to support a robust account based on Frambach *et al.*’s [26] quality criteria.

Quality criteria	Actions to support a strong account
Credibility	A range of perspectives is represented (‘triangulation’ for completeness) Public involvement activities Input from a multi-disciplinary team (gerontology, falls prevention, medical sociology, vision science) to increase analytical depth Plausibility of findings checked by a CBS support group Themes are substantiated by verbatim quotes
Transferability	Demographic data to enhance contextual depth Maximum variation sampling strategy Rich contextualised accounts Iterative data analysis Sample limitations identified and described
Dependability	‘Information power’ to inform sample size RTA: themes are rooted in the raw data All interview data analysed
Confirmability	K.F. kept a reflexive diary throughout the study and recorded analytical memos in Nvivo. K.F. critically reflected on how her assumptions may have influenced knowledge, including her previous employment as a ROVI, and methodological aspects (e.g. research versus therapeutic interview). Inclusion of contrary cases Peer debriefing Audit trail (NVivo)

### Public involvement

Public involvement activities were conducted to (i) confirm if the topic was relevant to people with CBS, (ii) seek guidance about inclusive recruitment and data collection methods and (iii) check the understandability/accessibility of study documents. Consultations were conducted face to face or by telephone with CBS support group members (*n* = 5), staff at Sheffield Royal Society for the Blind (*n* = 2), Esme’s Umbrella support group leaders (*n* = 5) and their group co-ordinator who lives with CBS.

## Results

Seventeen people with CBS participated in a telephone interview (mean 42 minutes) between February and March 2023. See Tables 2 and 3. Twelve professionals took part between February and May 2024 by telephone or Zoom (mean 34 minutes): three community falls prevention specialists, two eye clinic liaison officers (ECLO) and seven rehabilitation officers (visual impairment) (ROVI). See Table 4.

**Table 2:** Participants with CBS demographics.

Age	
50–59	2
60–69	3
70–79	4
80–89	5
≥90	3
Gender	
Female	10
Male	7
Ethnicity	
White British	17
Live alone	
Yes	6
No	11
Location	
Rural	4
Urban	13
Co-morbidities affecting mobility	
Yes	9
No	8
Main mobility aid used outdoors	
Support cane	5
Long cane	4
Guide cane	2
Symbol cane	2
Guide dog	1
Three wheel walker	1
Wheelchair	1
None	1
Formal mental health diagnosis	
Yes	2
No	15

**Table 3:** Clinical characteristics of participants with CBS.

Eye condition	
Age-related macular degeneration (neovascular or dry form)	7
Glaucoma	2
Inherited retinal disease	2
Multiple ocular pathologies	6
Certified as sight impaired	
Severely sight impaired	11
Sight impaired	2
Not registered	3
Declined registration	1
CBS diagnosis	
Formal diagnosis^a^	13
Self-diagnosis	4
Duration of CBS since symptom onset	
<1 year	1
1–2 years	3
3–4 years	5
5–6 years	5
7–8 years	1
≥9 years	1
Unable to recall	1
Frequency of CBS symptoms	
Intermittent (gaps in-between episodes of days or weeks)	10
Frequent (multiple time a day)	6
Continuous	1

^a^Formal diagnosis refers to a diagnosis by an eye health professional in primary or secondary care.

**Table 4:** Professionals’ characteristics.

Job role	Team	Participant code
Eye clinic liaison officer^a^	Hospital eye clinic/community outreach work	ECLO-1
Eye clinic liaison officer	Hospital eye clinic/community outreach work	ECLO-2
Senior physiotherapist	Community falls team	FP-1
Senior physiotherapist	Community falls team	FP-2
Senior occupational therapist	Community falls team	FP-3
Sensory team manager	Community sensory impairment team	STM-1
Rehabilitation officer (visual impairment)^b^	Third sector sight loss organisation	ROVI-1
Rehabilitation officer (visual impairment)	Community sensory impairment team	ROVI-2
Rehabilitation officer (visual impairment)	Community sensory impairment team	ROVI-3
Rehabilitation officer (visual impairment)	Third sector sight loss organisation	ROVI-4
Rehabilitation officer (visual impairment)	Community sensory impairment team	ROVI-5
Rehabilitation officer (visual impairment)	Community sensory impairment team	ROVI-6

^a^ECLOs provide practical and emotional support to eye clinic patients face to face or remotely and in the community.

^b^ROVIs, also known as vision rehabilitation specialists, support people with vision loss to maximise their independence in all areas of everyday life, including orientation and mobility skills

**Figure 1:**
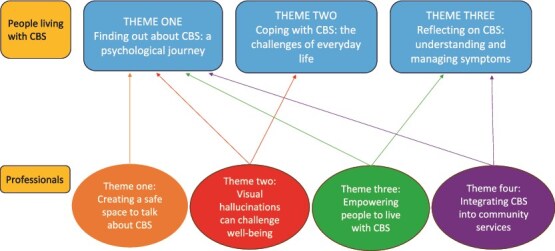
Mapping the themes.

Three themes were generated under the overarching concept of ‘the CBS journey’: (i) finding out about CBS: a psychological journey, (ii) coping with CBS: the challenges of everyday life and (iii) reflecting on CBS: understanding and managing symptoms. As professionals’ themes resonated with those from participants with CBS, findings were synthesised to provide a more comprehensive understanding. See Figure 1. The following section presents study findings supported by pseudonymised quotes. Codes for professionals are shown in Table 4.

### Theme one—finding out about CBS: a psychological journey

Theme one highlights the psychosocial challenges encountered pre to post CBS diagnosis. At symptom onset, most participants were unaware of CBS and did not automatically link experiences to vision loss:

At first I thought I were going doolally [Barbara]

Never been told about it, never met anybody that had it despite the fact I knew a few blind people [Paul]

I never thought it might be a crucial part of this thing that I’ve got [eye condition] [Debra]

My first reaction was well I’m getting dementia [Nancy]

Pathways to finding out about CBS were diverse and often convoluted: Barbara and Michelle classed their prompt diagnosis as ‘lucky’. The onus was on participants to disclose symptoms as eye health professionals seldom asked about CBS. Some informed the eye clinic team, but this was not always trouble-free: Jennifer was questioned by an ophthalmology registrar about her suicidal intent, and two participants felt upset when ophthalmologists who knew about CBS conveyed disinterest. A few spoke to a community optometrist about their symptoms, but their knowledge varied:

I didn’t know what it were, even down at optician didn’t know what it were when I told him [James]

Participants with persistent CBS sought a formal diagnosis, and for Jennifer, official recognition preserved a sense of self: ‘[I] could say to them [medics] when they said anything about mental health (…) I’ve got Charles Bonnet Syndrome’. A few with nondistressing symptoms self-diagnosed after discovering CBS via lay referral or sight loss charities.

Aftercare provided by eye clinics was rare but valued when offered, though information that did not resonate with personal experiences caused uncertainty. One participant was told that CBS images are always pleasant, and two people were informed that everyone sees ladies in period dress. Charles who ‘was very new to sight loss’ felt perplexed when an ECLO assumed he always retained full insight during a panoramic hallucination. Richard was upset by the insensitivity of wider healthcare professionals: ‘when I’m telling people about this [district nurse] they think it’s a huge joke’.

#### Professionals’ perspectives

Community sight loss professionals found that people rarely volunteered symptoms and created a safe space to talk about CBS. Lay language was favoured and most avoided ‘visual hallucinations’, classing it as a loaded term. ROVI-2 asked indirect, open questions, allowing people to raise CBS themselves and found more men than women divulged symptoms. Most asked directly: as STM-1 stated, ‘there’s a right time to bring it up’. Falls prevention specialists rarely encountered CBS but felt a script would be useful to prompt a discussion.

Contact with a community professional was largely referral-dependent. ROVIs visited when someone’s eyesight started to affect their functional independence; in the interim, some adopted a ‘forewarned is forearmed’ approach:

I’ll be invited along to the macular group (. . .) and as part of the talk I always bring up Charles Bonnet (. . .) anyone that might be sat there in silence (. . .) they don’t have to say anything but they get that reassurance [ROVI-3]

To prevent misdiagnosis, a few educated other teams, and some felt that mental health services and the police should be aware as they are privy to reports of ‘intruders’ in the home.

In most teams, CBS screening was not mandatory, and falls teams’ assessments were informed by NICE guidelines, which do not include CBS. All ROVIs made a ‘probable’ diagnosis and advised consulting a GP, but doubted CBS when symptoms deviated from published criteria, such as co-occurring auditory hallucinations.

### Theme two—coping with CBS and the challenges of everyday life

Theme two focuses on how CBS impacts everyday life. CBS interfered with mobility when it elicited a behavioural response:

I saw this cat right in the park in front of me (. . .) I had to lift my leg up and sort of try and climb over it and when I looked down there was no cat there [Jennifer]

Overriding automatic reactions to recognisable content was problematic:

[figures in a hospital corridor] they were walking straight at me (. . .) so I had to move to the right side of the corridor then to the left to dodge them [Mark]

[Little Red Riding Hood walking in front] I feel as if I’ve got to keep me distance, I don’t want to walk into this thing (. . .) but of course I know it’s not there [Debra]

[avoiding ‘birds] I’ll be walking then something will fly into me (. . .) I don’t even have time to think [Jennifer]

Participants who physically responded to CBS in public felt self-conscious:

I did halt for a few seconds [due to dancing figures] and then I thought well I’d better move or people will be thinking what’s wrong with her! [Barbara]

When CBS diverted attention or altered surroundings, participants felt disoriented, even in familiar environments:

[‘walls’ over doors at home] I get lost in the new architecture and I cannot figure what’s gone wrong or how to get out of it because everywhere I turn I’m blocked [Charles]

A few experienced a fall or near miss due to CBS. Michelle tripped over a kerb and fell while distracted by ‘all sorts of sparklies and all sorts of blobs’. Two people felt off balance when avoiding a hallucination, and Carol’s husband averted a fall after the ‘ground was just gone’. Charles highlighted how panoramic hallucinations influence decision-making about where to walk and that ‘there is no question of assessing the dangers’. Several participants felt CBS contributed to their concern about falling, including those with nonintrusive symptoms:

I have stopped walking because I think I’m a bit nervous at falling (. . .) on the pavement or on the floor [images] (. . .) I take care while I’m by myself [William]

If I’m walking about I’m very careful [Dorothy]

Navigating the environment with sight loss and co-occurring CBS was cognitively demanding. One participant with intrusive symptoms also negotiated real objects perceived differently:

[Yellow strips highlighting edge of steps] that [yellow strip] comes up to me it’s coming up and I can’t work [it] out so it’s really dangerous [Jennifer]

Long cane users found interpreting their surroundings alongside CBS exhausting. Akin to the fight or flight response, a couple of participants felt on high alert then emotionally drained after experiencing distressing CBS outdoors. Two people stopped using the bus due to CBS: one feared a spontaneous episode and the other always saw ‘men without faces’ dressed in black on board.

Simple hallucinations were mostly dismissed as a ‘nuisance’ or ‘annoyance’ but like the proverb ‘seeing is believing’, recognisable content was difficult to disregard:

[‘actors’ performing in home] It’s weird, it’s really foxed me a little bit because so much felt real [Richard]

they’re so real, everything is so real (. . .) it’s so real to the person who’s seeing it [Mark]

[children playing] they were so real so vivid [Debra]

James struggled to internalise a CBS diagnosis—“but I’m seeing them (…) I can see em, they’re there!”—and two people questioned their ability to accurately interpret surroundings. Even implausible content was not ignored: Debra touched ‘grass’ on a hospital floor for verification. Contextually appropriate images could delay insight until signs indicative of CBS became apparent:

This is strange the cat hasn’t moved because there were other people there (. . .) this really brave cat [Jennifer]

They never speak these people (. . .) there’s absolutely no sound (. . .) they look at you but their mouths don’t move (. . .) they suddenly disappear when they get into my eye line [Mark]

Most with intrusive CBS felt their symptoms remained the same or deteriorated (increased frequency, content became distressing). Some expressed an inability to escape and tried to reconceptualise CBS as their ‘new normal’:

[continuous CBS] I’ve had it for nearly 7 years (…) I’ve learnt to accept it (…) it’s part of my everyday life [Paul]

It’s hard but you just got to keep going haven’t you, there’s nothing else I can do [James]

#### Professionals’ perspectives

All professionals recognised CBS as a plausible contributor to falls. Two ROVIs met people who had stumbled, and others relayed fall incidents after someone jumped away and lost balance and following a collision during a panoramic hallucination. ROVI-2 was concerned about a carer’s safety:

If he stops suddenly [due to spontaneous CBS] that could bring both of them down

A few noted CBS restricted activity. For instance, ROVI-1 recalled an ‘experienced cane traveller’ who stopped going out as ‘every time he tried to (…) walk anywhere he was faced with a brick wall’. One professional met someone who had a permanent sighted guide as CBS changed familiar surroundings. Two ROVIs reported that sensations caused by CBS increased concern about falling:

He would say to me I know I’m in my living room but I feel I’m up in the air [ROVI-6]

Some ROVIs struggled to promote insight when people who were unaware of CBS responded to emotive, repetitive content such as a ‘colony of ants’. ROVI-5 recalled someone declining rehab due to CBS as they could not ‘get [their] head around everything that was happening’.

### Theme three—reflecting on CBS: understanding and managing symptoms

Theme three reflects the final phase of the CBS journey, specifically how people made sense of the images, precipitating factors and self-management. Although content was often deemed random, some linked it to interests and past experiences:

You might say why did you see mounted horse? Well I’ve lived near the football ground and on football day matches they deploy mounted police [Mark]

[describing part of a panoramic hallucination] it’s a scene that’s familiar to me from a painting I know and like [Charles]

Mark believed CBS was his ‘brain having fun’, and Jennifer felt her mind was ‘playing tricks’ when black fluff turned into spiders, and after moving closer, a ‘rhinoceros’ in a car park was in fact a covered motorbike.

Sense-making extended to why symptoms start and fluctuate. Participants with frequent or intermittent CBS struggled to identify triggers but reflecting on experiences proved insightful. Frequently reported exacerbating factors were a decline in vision, eye closure at night, dim light, being sedentary with a fixed gaze, tiredness, watching television, and travelling in a car. During the latter, content repeated and included scaffolding, a wall, hedges and a train; Richard saw ‘a heap of little children (…) a foot or two away from the front of the car’, which caused distress.

Participants with intrusive CBS also felt that heightened emotions were influential. Examples included loneliness, anxiety, depression, bereavement and pain and many identified situational stress:

[waiting for guide dog during a free run] for those seconds (…) that I can’t hear George my stress levels do rise (…) and the Charles Bonnet suddenly becomes much more prevalent [Paul]

If you’re having a really stressed day then the hallucinations are longer [Michelle]

One participant found disembodied faces become grotesque following a bereavement. A few believed mental health and personality traits predisposed someone to CBS.

Many found reactive relief techniques had limited utility:

I try to do the exercises, move my eyes from left to right and things like that and even getting up and walking out of the room. It doesn’t make them go away [Carol]

I’ve clapped me hands and that doesn’t work. I shout at them and that doesn’t work [Richard]

especially for those with distressing symptoms, although Charles found figures and panoramic hallucinations stopped after interacting with the image. Instead, indirect approaches were beneficial. Symptoms were absent or less intrusive (if continuous) while engaging in enjoyable activities such as gardening, cooking and listening to music. Many also found taking part in physical activity for health helpful, and undertook exercise in the community with friends and/or family, or attended accessible group-based classes:

[Exercising at local sight loss organisation] it’s not highly competitive (. . .) it’s fun, we go there for a laugh, enjoyment (. . .) we encourage each other, it’s a feel good factor [Mark]

To mitigate risks while walking, routes triggering CBS were avoided, and some stopped *en route* to relax or mentally adjust to changes in light. Participants working with a guide dog reported CBS was absent or less prominent, unlike when they used a long cane which was comparatively stressful.

Many valued the reassurance and sense of belonging CBS support groups provided, but Dorothy could not relate to others’ upsetting accounts and left after one session. Paul found shared experiences extended beyond human interaction:

[talking about guide dog] When I'm having a bad episode of Charles Bonnet (. . .) he will climb on me (. . .) put his paws on my shoulder (. . .) like he’s giving me a full-on cuddle (. . .) you would be surprised how comforting that is

Michelle employed both reactive and preventative management strategies: meditation, even home lighting and eccentric viewing technique to increase visual input.

#### Professionals’ perspectives

All professionals wanted sufficient CBS knowledge to fulfil their role. Falls specialists requested one off training while ROVIs sought an in-depth understanding with most feeling ill-equipped to help manage intrusive CBS. A few sight loss professionals highlighted that many relief strategies were unsuitable for people with no light perception, while ROVI-3 found rehabilitation approaches could be counter-productive:

strategies [optimal illumination] we teach to improve someone’s level of functional vision may actually result in someone suffering from Charles Bonnet, I’m convinced [ROVI-3]

Some professionals ran CBS groups, but others were unaware of this support network.

As CBS was rarely embedded into service provision, a few individuals drove changes in practice. Only one sensory team offered comprehensive CBS support: staff training, routine screening, a ‘CBS champion’ and specialist counselling for people with debilitating symptoms.

## Discussion

Identified themes highlight the physical, cognitive and psychosocial challenges CBS presents during the ‘CBS journey’, spanning symptom onset to living with CBS.

Although our study indicates CBS was most prevalent while sedentary—a finding reported elsewhere [27]—it could impede the ability to ‘move about’, reflecting cross-sectional data [15, 19]. Like other researchers [19, 28], we also found that hallucination content alone does not explain negative outcome CBS. Instead, other factors including the impact of CBS on daily activities are influential. Our findings help to illuminate how CBS can disrupt everyday life by compromising safety while moving about. We propose that four CBS characteristics can contribute individually or in different combinations to a cognitive-motor response that can exacerbate fall risk. (i) type of content, (ii) static or dynamic presentation, (iii) perceived proximity to self and (iv) spontaneity of presentation.

Participants’ accounts imply that simple, static images located ahead were easier to dismiss while recognisable stationary or dynamic content led to stepping actions in avoidance. Other studies also report people moving around images in their pathway [28, 29]. By contrast, static or dynamic complex images near the body, especially if spontaneous, were interpreted as an immediate threat and sudden protective movements compromised balance. Reactive balance—unlike anticipatory—renders proactive activation of trunk and leg muscles and postural adjustments impossible [30], increasing the risk of falling.

We found recognisable content held the gaze, and dynamic content (simple and complex) was distracting. Another qualitative study found CBS diverted attention during a road crossing [28]. We propose that CBS may interfere with visual attention by adding to ‘environmental clutter’. As the brain cannot process all incoming stimuli, we fixate on what is important [31]. To signal danger, the visual system is adept at processing facial expressions and movement [32]. Thus, dynamic faces and images—both reported by participants—may reduce the ability to monitor the environment.

Based on our data, panoramic hallucinations appear immersive and compelling and posed significant challenges to safety by causing disorientation, influencing decision-making about where to walk, and ‘obscuring’ real obstacles. Needham and Taylor [33] also reported someone falling down a stairway after avoiding a ‘cliff edge.’ Although more research is needed, whole scene hallucinations could be a feature of CBS: Makin et al [34] found this type was not reported by people with dementia.

Our findings also highlighted a dimension of content that may increase susceptibility to falls: insight. The crux of insight is that visions look real, but patients use logic to recognise that they are not. Some participants voiced signs indicative of CBS. Silent images and unusual behaviour have already been reported [16, 35]; however, clarity of content and fine detail [16] were not mentioned during our study. Of relevance, many stressed how the visions, even when contextually inappropriate, ‘felt real’, which may have delayed active comparison with usual vision. Complex hallucinations can be perceptually convincing [36] and difficult to disregard initially [5, 13, 28]. In fact, one CBS brain imaging study found visual areas involved in face recognition were activated during a hallucination of a face [37] perhaps explaining CBS’s persuasive nature. Thinking about images or ‘reality monitoring’ while walking entails simultaneous cognitive and motor involvement known as dual tasking. Due to limited cognitive resources, performance in either domain may therefore be compromised [38], potentially increasing fall risk.

An associated finding was professionals’ difficulty promoting insight when people who were unaware of CBS physically responded to repetitive emotive content. The presence of insight differentiates CBS from other conditions causing visual hallucinations. But it has been suggested that age-related structural changes may be implicated in CBS pathogenesis [39] while repeating images can create memory expectancies and recur [36]. Advanced knowledge about CBS may aid preservation of insight over time.

Falls-related variables exacerbated by CBS were also described by participants. Reflecting other work [40], many felt CBS increased their concern about falling, but our study adds that sensations elicited by CBS and content ‘altering’ the ground may be contributory. Participants also raised factors limiting physical activity including feelings of embarrassment after reacting to a hallucination in public.

Other aspects of CBS experiences that can be used to inform future falls prevention strategies were also highlighted in our study. Precipitating factors while moving about were mainly linked to the quality and quantity of visual input. Dim light is a known trigger [12, 15], but one ROVI found optimising home lighting to maximise use of residual vision while moving about immediately preceded CBS onset. A survey by Christoph *et al.* [12] also found low and bright light conditions to be causal. Fluctuations in light level hinders the use of remaining vision, and people with sight loss are especially prone to glare and poor light/dark adaptation. Another trigger reported in one other source [41] was being a passenger in a car or bus, and we found that it can limit the use of public transport which may have implications for accessing community and/or social activities. Of note, many participants had central vision loss, meaning that while seated in a fixed position, the ability to see objects directly ahead and use visual skills (e.g. scanning and tracking) was compromised. Professionals need to be aware of the interplay between environmental conditions, functional vision and CBS.

Some participants felt that tiredness exacerbated their symptoms and highlighted that navigating their surroundings with sight loss and CBS was mentally exhausting, especially when using a long cane. Interestingly, one person described the cognitive challenge of negotiating functional vision and CBS outdoors: judging the distance of a real object located nearby was problematic, whilst an object in the distance was perceived as something entirely different. The role of ‘visual misperception’ (seeing one thing as something else) due to incomplete sensory information [42] could be relevant to CBS.

As reported in the literature [13, 43], heightened emotions exacerbated CBS and could alter its characteristics. Situational stress was a particular problem while outdoors. Powers *et al.* [44] proposed that beliefs and emotions may ‘penetrate perception’, affecting what is seen. Our study indicates the wider context of CBS may also contribute to negative affect. The psychological protection of timely information is acknowledged [12, 19, 28] and supported by our work, as participants did not always intuitively link vision loss to CBS, and some lived with CBS in silence. Menon [5] also found that until prompted, patients remained reticent. Unless symptoms are voiced, or a behavioural response observed, CBS is ‘invisible.’ Due to stereotypes surrounding sight loss and visual hallucinations, people are potentially exposed to ‘double stigma’ [45], increasing reluctance to divulge.

Participants reported eye health professionals’ CBS awareness varied, and a reactive approach to diagnosis was practised, despite sight loss being a core criterion. Conversely, community sight loss specialists proactively raised CBS but contact often occurred later in the eye disease trajectory. ‘Waiting well’ with sight loss is advocated in the UK Eye Care Support Pathway [46]. Provision of CBS information by ‘first touch point’ professionals may therefore need to be extended to community optometrists and GPs. Inclusion of CBS in the vision component of multifactorial falls assessment would also be helpful.

Similar to other studies [12], relief strategies used during an active hallucination were not universally beneficial, but we found they were least effective for people with persistent CBS. Instead, personally meaningful, cognitively engaging multisensory activities nonreliant on vision had therapeutic effects. Jones *et al.* [43] reported that military veterans with CBS—a population vulnerable to mental health challenges—found diversion helpful. We also identified that accessible low to moderate intensity communal physical activity in a welcoming setting alleviated symptoms. Competing stimuli can suppress hallucinations [43] but feeling relaxed and socially connected may also be influential. Currently, therapeutic strategies are limited and rarely discussed with the patient [47, 48].

**Figure 2:**
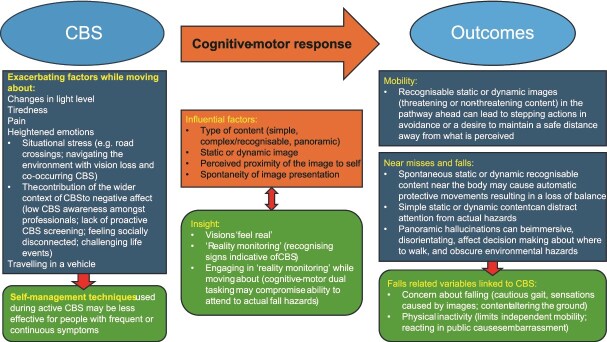
Conceptual figure: CBS and falls.

Findings showed that targeted psychosocial interventions were valued. CBS groups provided emotional support, and one sensory team offered counselling with positive outcomes. It has been suggested that CBS may be sustained by a negative feedback loop: hallucinations cause negative emotions, perpetuating symptoms [47]. However, CBS does not occur in a vacuum, and many participants faced trauma, including deteriorating vision and other significant life events. Specialist counselling merits investigation, but therapists need to be skilled in supporting people with sight loss and holistic approaches. A conceptual figure explaining CBS-related falls based on current available evidence is provided in Figure 2.

### Limitations

This study provides an in-depth understanding of CBS, physical activity and falls, and data quality was enhanced by public involvement, but there are limitations. All participants with CBS were white British and able to detect light or more, meaning experiences may differ from those of other ethnic groups and people with no perception of light. We recognise our recruitment criteria were broad but consider this a strength as it facilitated a more comprehensive understanding of CBS and falls. Exclusion of differential diagnoses for visual hallucinations was not feasible, but all participants demonstrated insight and capacity to consent. As professionals were self-selecting, their interest in CBS may not reflect the perspectives of colleagues. Only three falls prevention specialists contributed; however, the vision component in falls guidelines is broad, so we anticipated low CBS awareness. As the clinical magnitude of CBS and falls in the community is unknown, such data cannot be extrapolated to our findings.

## Conclusion

Medical case reports and surveys oversimplify CBS experiences. We suggest that CBS contributes to the ‘cognitive penalty’ of sight loss [49], as stereotypes surrounding visual hallucinations impact identity, and CBS contributes to cognitive burden by adding to vision loss and falls aetiology. Educating professionals about CBS, routine screening and provision of timely information may ameliorate the emotional impacts of CBS. However, the lack of evidence-based interventions limits support. When developing therapeutic strategies, wider impacts such as falls should be considered. CBS represents a journey but it is one journey that should not be travelled alone.

## Supplementary Material

afag125_Supplementary_materials

## Data Availability

Supplementary data mentioned in the text are available to subscribers in Age and Ageing online.

## References

[1] Mehta J, Baig A. The importance of assessing vision in falls management: a narrative review. Optom Vis Sci 2025;102:110–20. 10.1097/OPX.0000000000002222. 39960978 PMC11913239

[2] World Health Organization . World Report on Vision. https://www.who.int/docs/default-source/documents/publications/world-vision-report-accessible.pdf (17 November 2025, date last accessed).

[3] Forte G, Assaf N, Forte P et al. Charles Bonnet syndrome associated with unilateral vision loss: a new diagnostic perspective. Ophthalmic Physiol Opt 2025;45:681–8. 10.1111/opo.13481. 40099782 PMC11976511

[4] Subhi Y, Nielsen MA, Scott DAR et al. Prevalence of Charles Bonnet syndrome in low vision: a systematic review and meta-analysis. PROSPERO 2021;7:1–12. 10.21037/aes-21-37.

[5] Menon GJ . Complex visual hallucinations in the visually impaired: a structured history-taking approach. Arch Ophthalmol 2005;123:349–55. 10.1001/archopht.123.3.349. 15767477

[6] Burke W . The neural basis of Charles Bonnet hallucinations: a hypothesis. J Neurol Neurosurg Psychiatry 2002;73:535–41. 10.1136/jnnp.73.5.535. 12397147 PMC1738134

[7] Bridge H, Wyllie A, Kay A et al. Neurochemistry and functional connectivity in the brain of people with Charles onnet syndrome. Ther Adv Ophthalmol 2024;16:25158414241280201. 10.1177/25158414241280201. 39416975 PMC11481065

[8] daSilva MK, Collerton D, Firbank MJ et al. Visual cortical activity in Charles Bonnet syndrome: testing the deafferentation hypothesis. J Neurol 2025;272:199. 10.1007/s00415-024-12741-2. 39932561 PMC11813974

[9] Painter DR, Dwyer MF, Kamke MR et al. Stimulus-driven cortical hyperexcitability in individuals with Charles Bonnet hallucinations. Curr Biol 2018;28:3475–3480.e3. 10.1016/j.cub.2018.08.058. 30415703

[10] Jolly J, Assaf N, Higgins B et al. EEG changes associated with hallucinations caused by Charles Bonnet syndrome. Front Neurol 2025;16:1–7. 10.1016/j.cub.2018.08.058. PMC12815872 41567541

[11] Ffytche DH . Visual hallucinations and the Charles Bonnet syndrome. Curr Psychiatry Rep 2005;7:168–79. 10.1007/s11920-005-0050-3. 15935130

[12] Christoph SEG, Boden KT, Pütz A et al. Epidemiology and phenomenology of the Charles Bonnet syndrome in low-vision patients. Int Ophthalmol 2024;44:375. 10.1007/s10792-024-03298-0. 39256212 PMC11387450

[13] Vukicevic M . Frightening visual hallucinations: atypical presentation of Charles Bonnet syndrome triggered by the Black Saturday bushfires. Med J Aust 2010;193:181–2. 10.5694/j.1326-5377.2010.tb03843.x. 20678049

[14] Jones L, Ditzel-Finn L, Potts J et al. Exacerbation of visual hallucinations in Charles Bonnet syndrome due to the social implications of COVID-19. BMJ Open Ophthalmol 2021;6:e000670–0. 10.1136/bmjophth-2020-000670. PMC7880118 33628948

[15] Esteves Leandro J, Beato J, Pedrosa AC et al. Clinical characteristics of the Charles Bonnet syndrome in patients with neovascular-age related macular degeneration: the importance of early detection. Ophthalmic Res 2020;63:466–73. 10.1159/000506137. 31986513

[16] Kester EM . Charles Bonnet syndrome: case presentation and literature review. Optometry 2009;80:360–6. 10.1016/j.optm.2008.10.017. 19545849

[17] Teunisse RJ, Zitman FG, Cruysberg JRM et al. Visual hallucinations in psychologically normal people: Charles Bonnet’s syndrome. Lancet 1996;347:794–7. 10.1016/S0140-6736(96)90869-7. 8622335

[18] O'Brien J, Taylor JP, Ballard C et al. Visual hallucinations in neurological and ophthalmological disease: pathophysiology and management. J Neurol Neurosurg Psychiatry 2020;91:512–9. Review. 10.1136/jnnp-2019-322702. 32213570 PMC7231441

[19] Cox TM, Ffytche DH. Negative outcome Charles Bonnet syndrome. Br J Ophthalmol 2014;98:1236–9. 10.1136/bjophthalmol-2014-304920. 24825847 PMC4145458

[20] Fisher K, Sanders C, Stanmore E. Impact of Charles Bonnet syndrome on visually impaired older adults’ ability to engage in physical activity: a scoping review. Br J Vis Impair 2023;41:910–24. 10.1177/02646196221112800.

[21] Smith L, Timmis MA, Pardhan S et al. Physical inactivity in relation to self-rated eyesight: cross-sectional analysis from the English Longitudinal Study of Ageing. BMJ Open Ophthalmol 2017;1:e000046. 10.1136/bmjophth-2016-000046. PMC5751860 29354702

[22] White UE, Black AA, Delbaere K et al. Determinants of concern about falling in adults with age-related macular degeneration. Ophthalmic Physiol Opt 2021;41:245–54. 10.1111/opo.12777. 33368495

[23] Malterud K, Siersma V, Guassora AD Information power: Sample content and size in qualitative studies. In: PM Camic (Ed.), Qualitative research in psychology: Expanding perspectives in methodology and design (2nd ed., pp. 67–81). American Psychological Association. (2021). 10.1037/0000252-004

[24] Braun V, Clarke V. *Thematic Analysis: A Practical Guide*. Los Angeles: SAGE, 2022.

[25] V Braun, V Clarke. Understanding TA. University of Auckland. https://www.thematicanalysis.net/ (8 December 2022, date last accessed).

[26] Frambach JM, van der Vleuten CPM, Durning SJ. AM last page. Quality criteria in qualitative and quantitative research. Acad Med 2013;88:737–2. 10.1097/ACM.0b013e31828abf7f. 23531762

[27] Collerton D, Perry E, McKeith I. Why people see things that are not there: a novel perception and attention deficit model for recurrent complex visual hallucinations. Behav Brain Sci 2005;28:737–57. 10.1017/S0140525X05000130. 16372931

[28] Dave S, Jones L, Lee M et al. The experiences of visually impaired military veterans with Charles Bonnet syndrome. Ther Adv Ophthalmol 2024;16:25158414241294022. 10.1177/25158414241294022. 39524995 PMC11549713

[29] Paulig M, Mentrup H. Charles Bonnet's syndrome: complete remission of complex visual hallucinations treated by gabapentin. J Neurol Neurosurg Psychiatry 2001;70:813–4. 10.1136/jnnp.70.6.813. 11430294 PMC1737381

[30] Lee P-Y, Tsai Y-J, Liao Y-T et al. Reactive balance control in older adults with diabetes. Gait Posture 2018;61:67–72. 10.1016/j.gaitpost.2017.12.030. 29306146

[31] Boynton GM . Attention and visual perception. Curr Opin Neurobiol 2005;15:465–9. 10.1016/j.conb.2005.06.009. 16023853

[32] Snowden RJ, Thompson P, Troscianko T. *Basic Vision: An Introduction to Visual Perception*. Rev. edition. Oxford: Oxford University Press, 2012.

[33] Needham WE, Taylor RE. Benign visual hallucinations, or “phantom vision” in visually impaired and blind persons. J Vis Impair Blind 1992;86:245–8. 10.1177/0145482X9208600605.

[34] Makin SM, Redman J, Mosimann UP et al. Complex visual hallucinations and attentional performance in eye disease and dementia: a test of the perception and attention deficit model. Int J Geriatr Psychiatry 2013;28:1232–8. 10.1002/gps.3947. 23559442

[35] Menon GJ, Rahman I, Menon SJ et al. Complex visual hallucinations in the visually impaired: the Charles Bonnet syndrome. Surv Ophthalmol 2003;48:58–72. 10.1016/S0039-6257(02)00414-9. 12559327

[36] Collerton D, Barnes J, Diederich NJ et al. Understanding visual hallucinations: a new synthesis. Neurosci Biobehav Rev 2023;150:105208. 10.1016/j.neubiorev.2023.105208. 37141962

[37] Ffytche DH, Howard RJ, Brammer MJ et al. The anatomy of conscious vision: an fMRI study of visual hallucinations. Nat Neurosci 1998;1:738–42. 10.1038/3738. 10196592

[38] Pitts J, Singhal K, Apte Y et al. The effect of cognitive task, gait speed, and age on cognitive–motor interference during walking. Sensors (Basel) 2023;23:7368. 10.3390/s23177368. 37687823 PMC10490746

[39] Carpenter K, Jolly JK, Bridge H. The elephant in the room: understanding the pathogenesis of Charles Bonnet syndrome. Ophthalmic Physiol Opt 2019;39:414–21. 10.1111/opo.12645. 31591762

[40] Needham W, Taylor R. Atypical Charles Bonnet hallucinations: an elf in the woodshed, a spirit of evil, and the cowboy malefactors. J Nerv Ment Dis 2000;188:108–15. 10.1097/00005053-200002000-00007. 10695839

[41] Khan JC, Shahid H, Thurlby DA et al. Charles Bonnet syndrome in age-related macular degeneration: the nature and frequency of images in subjects with end-stage disease. Ophthalmic Epidemiol 2008;15:202–8. 10.1080/09286580801939320. 18569816

[42] Zavagno D . Illusion as a cognitive clash rooted in perception. J Intelligence 2023;11:215. 10.3390/jintelligence11110215. PMC10672324 37998714

[43] Jones L, Lee M, Ditzel-Finn L et al. Charles Bonnet syndrome among visually impaired military veterans: findings from a UK screening and survey study. BMJ Open Ophthalmol 2025;10:e001781. 10.1136/bmjophth-2024-001781. PMC11979505 40199613

[44] Powers A, Kelley M, Corlett PR. Hallucinations as top-down effects on perception. Biol Psychiatry: Cognit Neurosci Neuroimaging 2016;1:393–400. 10.1016/j.bpsc.2016.04.003. 28626813 PMC5469545

[45] Mula M, Kaufman KR. Double stigma in mental health: epilepsy and mental illness. BJPsych Open 2020;6:e72–2. 10.1192/bjo.2020.58. 32654672 PMC7443902

[46] Cooper R, Doyle H, Martin S. The UK Eye Care Support Pathway. London: RNIB, 2023.

[47] Baffour-Awuah KA, Bridge H, Engward H et al. The missing pieces: an investigation into the parallels between Charles Bonnet, phantom limb and tinnitus syndromes. Ther Adv Ophthalmol 2024;16:25158414241302065. 10.1177/25158414241302065. 39649951 PMC11624543

[48] Jones L, Jolly JK, Potts J et al. From research to action: recommendations for Charles Bonnet syndrome care and policy. BMJ Open Ophthalmol 2025;10:e002009. 10.1136/bmjophth-2024-002009. PMC12049992 40316414

[49] Ferrey AE, Moore L, Jolly JK. Renegotiating identity: the cognitive load of evaluating identity and self-presentation after vision loss. SSM Qual Res Health 2024;5:100372. 10.1016/j.ssmqr.2023.100372.

