# Supplementary material for: ‘Everywhere I turn, I’m blocked’: a qualitative exploration of experiences of Charles Bonnet syndrome and its impact on physical activity and falls
Source: Age Ageing. 2026 May 17;55(5):afag125. doi: 10.1093/ageing/afag125 (PMC13180270; doi:10.1093/ageing/afag125)
Supplement: afag125_Supplementary_materials [file afag125_supplementary_materials.docx]

## “Everywhere I turn, I’m blocked”: a qualitative exploration of experiences of Charles Bonnet Syndrome and its impact on physical activity and falls

### Appendix 1: Interview guide - participants living with CBS:

**Explain the following prior to commencing the interview:**

- Can withdraw at any time.
- Can stop and have a break or continue the interview on a different day if find tiring.
- Remind not to share any information such as name and location that may identify them to help uphold confidentiality.
- Remind that participants should only give information that they feel comfortable sharing and that they do not have to answer any questions that they do not wish to.
- The interview will be recorded (state when the recorder is switched on/off).

***PART ONE: General questions about CBS and how it affects ability to carry out daily physical activities.***

1. **Please can you tell me about your experience of CBS.**

**Prompts:**

1. Moving around
   - Stop suddenly?
   - Step aside to avoid?
   - Distracted from real hazards?
   - Can you always tell the difference between real and hallucinatory objects?
   - Scared of falling
   - Fallen
2. Emotional/psychological impact

- Scared
- Don’t want to go out, feel reclusive

1. Exhaustion
2. Difficulty concentrating
3. **Is there anything that affects the frequency or content of hallucinations that you see?**

**Prompts:**

- Feeling anxious or stressed
- Travelling in a car
- Social isolation
- Inactivity
- Walking in unfamiliar areas

Is there anything that you have found to help stop the visions, or change what you see?

**Prompts:**

- Meditation, relaxation techniques
- Exercise
- Being outdoors
- Being distracted

**PART TWO – Focused questions about engaging in physical activity for health and strength/balance/flexibility.**

1. Are you aware of the Chief Medical Officer’s Physical Activity for health Guidelines? If yes, can you recall what they are?

If no, read the following:

Read aloud:

*It is recommended that people aged 18 years and over take part in 2 ½ hours of moderate intensity aerobic exercise each week such as a brisk walk, carrying shopping bags, dancing and cycling.*

*It is also recommended that people aged 65 and over carry out activities to increase muscle strength, balance and flexibility twice a week. Examples include yoga, pilates, heavy gardening, carrying shopping bags.*

1. Which physical activities for health do you take part in? Ask for examples
2. Are there any physical activities for health that you would you like to take part in? Ask for examples
3. What might stop you from taking part in physical activities for health?

****CLOSE****

## “Everywhere I turn, I’m blocked”: a qualitative exploration of experiences of Charles Bonnet Syndrome and its impact on physical activity and falls

### Appendix 2: Coding list and development of themes – participants living with CBS

**RTA = Reflexive Thematic Analysis**

**Codes (phases one and two of RTA)**

Low CBS awareness (patients, professionals)

Spontaneous onset

What is happening to me?

Onus on individual to disclose

Low awareness of functional impacts of CBS (professionals)

Psychosocial impacts

CBS characteristics

It “feels real”

Automatic response

Signs indicative of CBS

Cognitive-behavioural responses (to CBS)

CBS interferes with mobility

CBS is disorientating

CBS causes near misses

CBS and falls

Concern about falling

Activity restriction

Feel embarrassed reacting in public

Navigating the environment with CBS is exhausting

Disentangling CBS experiences

Predisposing factors

Precipitating factors (personal, wider context)

Variable success of reactive relief techniques

Feeling trapped (uncontrolled symptoms)

Accessible physical activity

Therapeutic effects of physical activity

Therapeutic effects of diversion

A toolkit of strategies (reactive, preventative)

**Candidate themes (phase three of RTA)**

**Theme 1:** What is happening to me?

**Theme 2:** The functional impacts of CBS

**Theme 3:** Engaging in physical activity

**Theme 4:** Starting to make sense of what’s happening

**Theme 5:** Reigning in CBS symptoms

**Final themes (phase four of RTA)**

**Theme 1:** Finding out about CBS: a psychological journey

**Theme 2:** Coping with CBS: the challenges of everyday life

**Theme 3:** Reflecting on CBS: understanding and managing symptoms
